# Multiple Levels of Chemokine Receptor Regulation in the Control of Mouse Natural Killer Cell Development

**DOI:** 10.3389/fimmu.2014.00044

**Published:** 2014-02-13

**Authors:** Giovanni Bernardini, Giorgia Benigni, Fabrizio Antonangeli, Andrea Ponzetta, Angela Santoni

**Affiliations:** ^1^Laboratory of Molecular Immunology and Immunopathology, Department of Molecular Medicine, Istituto Pasteur-Fondazione Cenci Bolognetti, “Sapienza” University of Rome, Rome, Italy; ^2^Neuromed, Pozzilli, Italy

**Keywords:** NK cell, chemokine receptor, transcription factors, microRNA, lymphocyte development and function, bone marrow

## Abstract

Chemokines play a fundamental role in lymphocyte development, mainly attributable to the control of the correct localization in the proper microenvironments of cells undergoing maturation. Natural killer (NK) cell development occurs in the bone marrow (BM) where their localization is regulated by the balance of chemokine function in cell retention into tissues and mobilization into circulation. In addition, NK cells from several extra-medullary tissues are phenotypically and functionally different from their circulating counterpart suggesting that maturation can be completed in organs other than BM. Indeed, a role of chemokines in NK cell localization into tissues during homeostatic conditions is also documented. In this review, we summarize the current notion related to the relevance of several chemokine/chemokine receptor axes in NK cell development with a focus on the regulation of their expression and function.

## General Introduction on Natural Killer Cell Development

Natural killer (NK) cells are innate lymphocytes that provide host protection against infectious diseases and cancer. NK cells recognize ligands expressed by infected and transformed cells through germline encoded activating receptors resulting in the killing of target cells and production of immunomodulatory cytokines (1).

Bone marrow (BM) represents the main site for NK cell development, providing a full array of stimuli organized in complex microenvironments and acting in concert to sustain NK cell differentiation. Starting from the classical distinction of a committed precursor (NKP: Lin^−^/CD122^+^/NK1.1^−^/CD49b^−^), an immature (iNK: NK1.1^+^/CD49b^−^CD11b^low^/CD51^low^) and a mature NK subpopulation (mNK: CD49b^+^/Ly49s^+^), the NK cell developmental process has been progressively dissected through a more detailed comprehension of the great cell diversity within each stage (2–4). Recent findings better characterize the lineage commitment process, with the identification of an earliest NK cell progenitor in mouse BM that links the CLP and the NKP (5, 6), and is characterized by the expression of the surface molecules CD127 (IL-7 receptor), CD244, CD27, and of the basic helix-loop-helix transcription factor (TF) Id2.

A key marker of the NK cell lineage is CD27, a member of the TNF receptor superfamily, whose expression, combined with the integrin chain CD11b, defines four sequential developmental stages: CD11b^low^CD27^low^, CD11b^low^CD27^high^, CD11b^high^CD27^high^, and CD11b^high^CD27^low^ subsets (7). As a final step, the most mature subset acquires the expression of the inhibitory receptor KLRG1 that defines a cell subset with reduced effector functions, decreased IL-15 responsiveness, and proliferative capacity (8). However, our group demonstrated that also this terminally differentiated subset is heterogeneous, since the expression of the chemokine receptor CX3CR1 characterizes an even later maturation stage with unique functional features and localization within BM microenvironments (9).

## Tissue-Specific NK Cell Maturation

**Figure 1:**
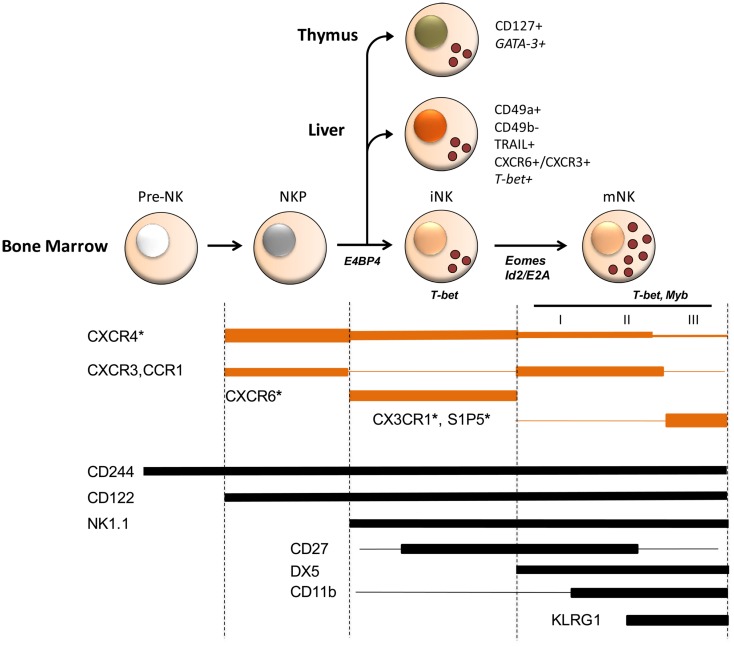
**Schematic model of chemoattractant receptor expression during mouse NK cell development and of transcription factors involved in NK cell commitment and maturation**. Several stages of differentiation have been defined during which NK cells progressively acquire maturation markers (2–4). Three stages (I–III) corresponding to different levels of functional competence were described in mature CD49b^+^ (DX5^+^) NK cells, being CD27^high^CD11b^low^ and CD11b^high^CD27^high^ fully competent and a later stage of differentiation marked by the inhibitory receptor KLRG1 having reduced functional capacity. Besides mNK, iNK are found in peripheral tissues, such as liver and thymus, where they represent NK cells with tissue-specific phenotype and functions possibly arising from a BM precursor. Chemokine and S1P receptor expression is developmentally regulated and is associated to selective functions in NK cell localization and maturation in BM. For example, CX3CR1 and S1P_5_ play an important and non-redundant role in mature NK cell egress from BM. Although, it is not known whether these two receptors are co-expressed by the same cell or expressed by different cell populations, prompting an investigation on how they co-operate to allow the efficient export of NK cells into circulation. Chemokine receptor expression overlaps that of several transcription factors active during NK cell development. Indeed some of these transcription factors, including T-bet and Myb were shown to directly affect chemokine and S1P receptor expression. Shown are transcription factors at the stage where they play a role in development. Asterisks identify chemoattractant receptors whose deficiency affects NK cell development. Line width is proportional to expression levels.

It is important to note that, beside the BM-driven maturation process, also extra-medullary sites contribute to guide NK cell development, thus generating tissue-specific NK cell subsets (Figure 1). NKPs have been found in murine thymus, spleen, liver, and lymph nodes (LN), but it is still unknown whether these cells are generated *in situ* or are recruited from BM. Hepatic CD49b^−^NK cells uniquely express the integrin chain CD49a and display the TNF-related apoptosis-inducing ligand (TRAIL), through which they can trigger cell death in susceptible targets (10–12). Although liver NK cell phenotype resembles that of immature cells, accumulating evidence suggest that CD49a^+^CD49b^−^ hepatic NK cells represent a resident population, able to mediate memory responses, and correspond to a different subset compared to BM iNK, whereas CD49a^−^CD49b^+^ hepatic NK cells are a migratory subset that mostly originate outside the liver (12). More controversial is the stability of the CD49b^−^ hepatic subset, as adoptive transfer experiments allowed some groups to demonstrate that this population can repopulate several organs and give rise to CD49b^+^ cells (10, 13), while others evidenced that CD49b^−^ cells are a stable subset that can only repopulate liver (12). An alternative pathway of NK cell development takes place in the thymus (14), generating a phenotypically and functionally distinct subset characterized by the expression of the α-chain of CD127 and high amounts of the TF GATA-3. The authors reported that CD127^+^ NK cells also constitute a relevant fraction of NK cells in LN that may arise from thymus due to their CD11b^low^CD16^−^CD69^high^Ly49^low^ phenotype and to their selective reduction in athymic nude *Foxn*^−/−^ mice. Nevertheless, other authors found normal number of CD127^+^ NK cells in LN of *Foxn*^−/−^ mice (15).

## Regulation of Gene Expression in Developing NK Cells

Although the differential expression of maturation-related surface markers can help to distinguish different stages of development, the precise sequence and combination of stimuli controlling NK cell acquisition of functional properties, organ-specific features, inter-organ and intra-organ positioning, and chemokine receptor expression require further investigation. One critical aspect related to NK cell development is the transcriptional and post-transcriptional regulation of mRNA expression of molecules associated with developing NK cell phenotype and function. Several TFs and, more recently, selected microRNA (miRNA) expression were shown to be involved in the commitment to the NK cell lineage and in NK cell maturation (16–18) (Figure 1).

E4-binding protein 4 (E4BP4), is a basic leucine zipper (bZIP) TF required for NK cell development and maturation as its deficiency leads to marked reduction of iNK and lack of mNK, without affecting other hematopoietic cell lineages (19). E4BP4 promotes the expression of Id2 and GATA-3, and forced expression of Id2 in E4BP4-deficient mice partially rescued the NK cell deficiency. Nevertheless, Id2 plays a critical role only for differentiation to mNK (19, 20). Gordon et al. demonstrated that the shift from immature TRAIL^+^CD11b^low^ to CD49b^+^CD11b^hi^ NK cells and the parallel induction of Ly49 receptors in BM and liver require the expression of Eomesodermin (Eomes), a member of the T-box family of TFs (13). In this regard, a subset of less mature TRAIL^+^ NK cells that lacks CD49b and Eomes expression is expanded in neonates, and preferentially resides in adult liver (10, 21). It has been proposed that Eomes-dependent maturation is restricted by the TF T-bet in Eomes^−^CD49b^−^ iNK as NK cells with T-bet deletion exhibit increased levels of Eomes expression and accelerated maturation (13). Nevertheless, T-bet is still expressed in Eomes^+^ mature NK cells, suggesting that other factors expressed in mNK cells circumvent T-bet to up-regulate Eomes expression (22).

Concerning miRNAs, conditional knockdown of the miRNA-processing enzyme Dicer, which leads to a global impairment of the miRNA biogenesis, results in decreased mature NK cell number due to increased apoptosis of peripheral NK cells and accumulation of immature NK cells (18). Accordingly, significant accumulation of more immature CD27^high^CD11b^low^ and a related decrease of IFN-γ production have been observed also within miRNA-150^−/−^ NK cells (23). This was associated to higher stability of the TF c-Myb, a direct target of miRNA-150 negatively regulated during the transition from CD27^high^CD11b^low^ to CD27^low^CD11b^high^ NK cells (7). Similarly, miRNA-155 has been shown to enhance IFN-γ production in human NK cells activated by IL-2/IL-18 (24). Differently from miRNA-150, in miRNA-155^−/−^ mice, the immature CD27^high^CD11b^low^Ly49D^−^Ly49H^−^NK cell subset is reduced, likely because miRNA-155 targets the pro-apoptotic factor Noxa, thus supporting survival and homeostasis of developing NK cells (25). In human, miRNA-181 expression increases during NK cell maturation and promotes NK cell differentiation by regulating Notch signaling, essential for lymphocyte development (26).

## Chemokines and Lymphocyte Development

Chemokines are small cytokines with pleiotropic functions, having effect on a broad range of leukocytes and are thus critical regulators of immune cell function (27). Based on the presence of conserved cysteine residues, there are two major (CXC and CC) and two minor (C and CX3C) chemokine classes and accordingly four classes of chemokine receptors (CXCR, CCR, CX3CR, XCR). Another chemoattractant, the lipid sphingosine-1-phosphate (S1P) and its receptors (S1P_1–5_) are required for lymphocyte egress from lymphoid organs (28).

The chemokine system regulates the process of lymphocyte generation, mainly by directing developing cell positioning in successive compartmental niches, which provides exposure to specialized microenvironments in primary lymphoid organs. The leading chemokine/chemokine receptor axis in hematopoiesis is CXCL12/CXCR4, which regulates hematopoietic stem and progenitor cell homing to BM, retention in specialized niches, proliferation, and egress to the circulation (29, 30).

In regard to B cell lymphopoiesis, CXCL12/CXCR4 axis is widely known to be crucial to maintain homeostatic levels of B cell precursors in the proper BM anatomical sites (31–33). More recently, a finely regulated post-receptor signaling was also reported: *in vitro* long-term exposure of pro-B cells to CXCL12 induces their strong and sustained adhesion to VCAM-1, resulting in prolonged CXCL12-induced focal adhesion kinase (FAK) phosphorylation in immature cells, and promotion of progenitor cell growth, survival, and differentiation (34, 35). As long as cells differentiate, CXCL12-induced FAK phosphorylation becomes short-lived, decreasing adhesiveness of mature cells, and enhanced exit in peripheral circulation. Besides this, changes in scaffold proteins in cytosol or membrane and in the glycosylation pattern of CXCR4 occur during B cell maturation (36).

Concerning T cell maturation, it is well-established that CLPs generated in BM reach the thymus through venules in the cortico-medullary junctions, where their maturation process is regulated by chemokine-driven migration from medulla toward outer cortex and subcapsular zone, with a leading role for CCL19–CCL21/CCR7, CCL25/CCR9, and CXCL12/CXCR4 axes (37). In addition, CXCR4 acts on early thymocyte development as co-stimulator of the pre-TCR, providing MAPK and PI3K-dependent survival signals, and promoting the double negative (DN)3 to DN4 transition (38, 39). Recently, a crucial role in T cell selection and development for the atypical receptor CCX-CKR has also been documented, linked to its function as decoy/scavenger receptor for CCR7 and CCR9 ligands (40).

In regard to NKT cells, development starts in thymus and is completed in peripheral tissues, mainly in liver, where an important role was recently attributed to CXCL16/CXCR6 axis. Absence of CXCR6 leads to reduced number of mature NKT cells in liver and accumulation of immature cells in BM and spleen, due to altered trafficking and impaired maturation of thymus-derived cells (41).

## Regulation of Chemokine Receptor Expression in NK Cell Development

Accumulating evidence indicates that the chemokine system can influence NK cell development through the regulation of several aspects of NK cell biology (42). NK cells change their chemokine receptor expression profile during development in BM (Figure 1). CXCR4 is highly expressed by NKP but its expression progressively decreases on iNK and mNK. On the other hand, CXCR3 and CCR1 are up-regulated on CD49b^+^KLRG1^−^mNK. CX3CR1 and the chemoattractant receptor S1P_5_ are prevalently present on more differentiated NK cells, being the expression of CX3CR1 mainly confined to the KLRG1^+^ subset that poorly expresses CXCR4 and CXCR3 (9, 43, 44). CXCR6 is expressed only by immature cells, a phenotype that is maintained also by liver resident CD49b^−^Ly49^−^ NK cells.

NK cell development is severely impaired in CXCR4 conditionally deficient adult mice where NK cells are markedly reduced in number and display reduced cytotoxic function and IFN-γ production capacity (45). The defect was associated with reduced number of NK cell precursors and decreased proliferation rate of CXCR4 deficient iNK. Of note, this effect could be related to regulation of developing NK cell maintenance into maturation niches, as we previously demonstrated that CXCR4 differentially affects NK cell retention into BM according to their maturation stage (9, 44). Accordingly, transient CXCR4 desensitization within BM promotes NK cell exit, likely facilitating the mobilizing effect of other chemoattractant receptors (46). In this regard, it was demonstrated that a fraction of CXCL12 abundant reticular (CAR) cells that express high levels of CXCL12, co-express IL-15 and IL-15Ralpha and can be found in close proximity with NK cells *in vivo*, thus suggesting that CAR cells trans-present IL-15 and provide CXCL12 to NK cells during development (45). The same authors also evidenced that CXCL12 enhances the effect of IL-15 on mNK cell generation from lymphoid precursors and iNK cells *in vitro*, suggesting that CXCR4 acts as a co-stimulus during NK cell differentiation.

Reduced NK cell number in several tissue compartments and impaired killing capacity of NK cells were also evidenced in CCR5 but not in CCR1 deficient mice under normal conditions. The defect was attributed to a slight reduction of NK cell precursor number and to a lower *in vivo* proliferation capacity of CCR5 deficient NK cells. The authors suggested that reduced number of proliferating NK cells may result either from incorrect localization of these cells in their specific niche or from deficiency in co-stimulatory signals provided by CCR5 (47).

Because CX3CR1 is expressed by a small fraction of mouse NK cells, to evaluate its role under physiological conditions we recently made use of a CX3CR1–GFP reporter mouse, where GFP is expressed under the control of CX3CR1 promoter. The subset identified by means of GFP expression, was expanded in all analyzed tissues of CX3CR1-deficient mice indicating that CX3CR1 regulates the homeostatic number of NK cells (48). A role of the CX3CR1 in NK cell maturation was also suggested, as NK cells from CX3CR1-deficient mice displayed a more pronounced degranulation capacity and mobilization from BM into blood, respectively after *in vitro* and *in vivo* activation. The increased number of CX3CR1-deficient NK cells in BM could be associated to their reduced exit in circulation by competitive adoptive transfer experiments. Similarly, S1P_5_ provides an egress signal to NK cells, allowing their export from the BM and the LNs (43).

Using CXCR6–GFP reporter mice, Paust et al. demonstrated that CXCR6 is important for the development and/or survival of CXCR6^+^ NK cells exclusively in the liver. CXCR6^−/−^mice have significantly reduced GFP^+^ cells in liver, compared to CXCR6^+/−^mice, but normal number of GFP^−^ cells. The authors were able to show that the phenotype observed is not due to altered trafficking, but is likely due to the absence of CXCR6-mediated NK cell localization/survival in sinusoidal endothelium, which constitutively presents CXCL16 (49).

How are the effects of multiple chemokine receptors regulated, to finely tune the different steps of NK cell maturation? One level of control is the differential expression of chemotactic ligands by BM cellular niches. Of note, beside CXCL12, other chemokines are expressed in BM, although their cellular sources are less clear. Human BM stromal cells express CCL2, CX3CL1, and CXCL8, and, more specifically, osteoclasts can produce CCL5 and CCL9 during steady state (50, 51). A similar pattern of chemokine expression can be detected in mouse BM (48, 52). However, responsiveness to the chemokine environment requires an efficient control of chemotactic receptor gene expression, which represents a second mechanism of regulation. Interestingly, miR-150 knockdown significantly increased CXCR4 expression in mononuclear hematopoietic cells and affected their BM localization (53). It is likely that the effect of miR-150 on CXCR4 mRNA expression is Myb-dependent as this TF can associate the CXCR4 gene promoter and activate a CXCR4 reporter gene in transfection assays. Similarly to CXCR4, Myb expression decreases during NK cell maturation paralleling the increased expression of miR-150, thus suggesting that reciprocal regulation of these three factors plays a critical role in NK development during homeostasis. Regulation of CXCR4 function by miRNAs may also occur at the signaling level as another regulator of NK cell development, miR-181, was shown to repress PTEN expression in NKT cells thus allowing proper CXCL12-stimulation of Akt without affecting CXCR4 expression during thymic development (54).

Regulation of chemoattractant receptor expression by TFs in developing NK cells was also documented. T-bet promotes expression of S1P_5_ but not S1P_1_ by mNK, and its deficiency leads to impaired CXCR3 expression (55). Interestingly, Eomes^−^ and Eomes^+^ NK cells express different repertoires of homing receptors consistent with their preferential anatomic localization (liver versus BM respectively) thus allowing the correct maturation of liver versus BM NK cell populations (13). Indeed, CD49b^−^Eomes^−^ NK cells expressed the integrin chain α_v_ and the chemokine receptors CXCR3 and CXCR6 at greater levels relative to Eomes^+^ NK cells and expressed substantially lower levels of the S1P receptors S1P1 and S1P5 than Eomes^+^ NK cells. As we could not detect CXCR3 on CD49b^−^ (DX5^−^) NK cells in BM and spleen, expression of CXCR3 by this subset may be a unique property of hepatic NK cells (44). CXCR3 and CXCR6 expression in liver was linked to Eomes expression as repression of this TF caused re-expression of these chemokine receptors. Thus, Eomes and T-bet may regulate the differential expression of chemokine receptors at different stages of NK cell development.

ID proteins are essential for NK cell development and maturation, thanks to their ability to inhibit E-protein and in particular E2A, a family of TFs that act as transcription activators or repressors (20, 56). Id2 and E2A have been suggested to regulate emigration of NK cells from the BM as Id2^−/−^E2A^−/−^ mNK number was reduced in peripheral blood but was normal in BM. Although in Id2^−/−^E2A^−/−^mice, mNK fail to express the adhesion molecule CD11b, no defect in chemokine receptor expression, i.e., CCR1, CCR5, or CX3CR1 could be observed. Nevertheless, a regulation of chemokine receptor expression by E2A is possible as CCR7 expression may depend on ID protein-mediated E-protein down-regulation in thymocytes (57). Although mouse NK cells do not express CCR7, this mechanism of transcription may be relevant in humans where CCR7 plays a role in the localization of the NK cells in LN.

All together the studies presented herein evidence an emerging role of chemoattractant receptors in NK cell development. Thus, the identification of the molecular factors that regulate expression and function of these receptors and of BM niches where they can find and respond to their respective ligands will undoubtedly expand our comprehension of NK cell biology in the future.

## Conflict of Interest Statement

The authors declare that the research was conducted in the absence of any commercial or financial relationships that could be construed as a potential conflict of interest.
